# A novel interpretable deep learning model for diagnosis in emergency department dyspnoea patients based on complete data from an entire health care system

**DOI:** 10.1371/journal.pone.0311081

**Published:** 2024-12-27

**Authors:** Ellen T. Heyman, Awais Ashfaq, Ulf Ekelund, Mattias Ohlsson, Jonas Björk, Ardavan M. Khoshnood, Markus Lingman

**Affiliations:** 1 Department of Emergency Medicine, Halland Hospital, Region Halland, Sweden; 2 Emergency Medicine, Department of Clinical Sciences Lund, Lund University, Lund, Sweden; 3 Halland Hospital, Region Halland, Sweden; 4 Center for Applied Intelligent Systems Research (CAISR), Halmstad University, Halmstad, Sweden; 5 Skåne University Hospital, Lund, Sweden; 6 Centre for Environmental and Climate Science, Lund University, Lund, Sweden; 7 Division of Occupational and Environmental Medicine, Department of Laboratory Medicine, Lund University, Lund, Sweden; 8 Clinical Studies Sweden, Forum South, Skåne University Hospital, Lund, Sweden; 9 Emergency Medicine, Department of Clinical Sciences Malmö, Lund University, Lund, Sweden; 10 Skåne University Hospital, Malmö, Sweden; 11 Department of Molecular and Clinical Medicine, Institute of Medicine, Sahlgrenska Academy, University of Gothenburg, Gothenburg, Sweden; Tehran University of Medical Sciences, ISLAMIC REPUBLIC OF IRAN

## Abstract

**Background:**

Dyspnoea is one of the emergency department’s (ED) most common and deadly chief complaints, but frequently misdiagnosed and mistreated. We aimed to design a diagnostic decision support which classifies dyspnoeic ED visits into acute heart failure (AHF), exacerbation of chronic obstructive pulmonary disease (eCOPD), pneumonia and “other diagnoses” by using deep learning and complete, unselected data from an entire regional health care system.

**Methods:**

In this cross-sectional study, we included all dyspnoeic ED visits of patients ≥ 18 years of age at the two EDs in the region of Halland, Sweden, 07/01/2017–12/31/2019. Data from the complete regional health care system within five years prior to the ED visit were analysed. Gold standard diagnoses were defined as the subsequent in-hospital or ED discharge notes, and a subsample was manually reviewed by emergency medicine experts. A novel deep learning model, *the clinical attention-based recurrent encoder network* (CareNet), was developed. Cohort performance was compared to a simpler CatBoost model. A list of all variables and their importance for diagnosis was created. For each unique patient visit, the model selected the most important variables, analysed them and presented them to the clinician interpretably by taking event time and clinical context into account. AUROC, sensitivity and specificity were compared.

**Findings:**

The most prevalent diagnoses among the 10,315 dyspnoeic ED visits were AHF (15.5%), eCOPD (14.0%) and pneumonia (13.3%). Median number of unique events, i.e., registered clinical data with time stamps, per ED visit was 1,095 (IQR 459–2,310). CareNet median AUROC was 87.0%, substantially higher than the CatBoost model´s (81.4%). CareNet median sensitivity for AHF, eCOPD, and pneumonia was 74.5%, 92.6%, and 54.1%, respectively, with a specificity set above 75.0, slightly inferior to that of the CatBoost baseline model. The model assembled a list of 1,596 variables by importance for diagnosis, on top were prior diagnoses of heart failure or COPD, daily smoking, atrial fibrillation/flutter, life management difficulties and maternity care. Each patient visit received their own unique attention plot, graphically displaying important clinical events for the diagnosis.

**Interpretation:**

We designed a novel interpretable deep learning model for diagnosis in emergency department dyspnoea patients by analysing unselected data from a complete regional health care system.

## Introduction

Patients with dyspnoea have a higher short-term mortality than most other patients in the emergency department (ED) [1–3], therefore, an early diagnosis and treatment is essential [4–6]. The most prevalent diagnoses are acute heart failure (AHF), exacerbation of chronic obstructive pulmonary disease (eCOPD) and pneumonia, making up approximately half of dyspnoeic adults in emergency care [7]. These diagnoses are often mistaken for each other in the ED [8].

Studies indicate that final ED diagnoses, after standard ED evaluation, are concordant with the hospital discharge summary in 54–88% of AHF patients, 56–67% of eCOPD patients and 47–67% of pneumonia patients [9, 10]. Older dyspnoea patients receive correct treatment in the ED in only 64%, 54% and 68% of the AHF, eCOPD and pneumonia cases, respectively [10]. Inaccurate diagnostics have been correlated with higher mortality [5]. The diagnostic accuracy of dyspnoea has not improved over the last decades, as acute coronary syndrome, stroke/intracerebral bleeding and sepsis have; therefore, diagnostic decision support is urgently required [11].

Several artificial intelligence (AI) models for emergency diagnostics have outperformed standard care [12], but to our knowledge, there are no models diagnosing dyspnoea.

In this study, we aimed to create an AI diagnostic decision support for dyspnoeic adults at time of ED triage. Our approach was to analyse comprehensive and unselected real-world administrative and clinical data from an entire regional health care system in an open-ended search for diagnostic predictors and to present the result to the clinician in an interpretable way for each individual patient.

## Methods

### Setting

Region Halland, a region in southwestern Sweden, hosted this research. The region´s two hospital EDs serve 330,000 inhabitants annually with 46,000 and 42,000 ED visits, respectively.

### The cohort

This population-based cross-sectional study included all adult (≥ 18 years of age) ED visits with dyspnoea, i.e. the subjective experience of breathing discomfort, as the main complaint within the region´s two EDs from July 1, 2017, to December 31, 2019 (Table 1). The five-level Rapid Emergency Triage and Treatment System (RETTS) [13], Sweden’s most common triage system, was used to define the complaint. Dyspnoea as a chief complaint accounted for 6.9% of all adult ED visits. Patients referred to other levels of care immediately at triage, patients who left without being seen by a doctor (LWBS) and residents of other Swedish regions and other countries were excluded.

**Table 1:** Cohort selection.

Inclusion/exclusion criteria	Change (N)	Cohort size (N)
**Number of ED visits registered upon arrival 1st of July 2017–31st of December 2019**	N/A	221,208
**Number of ED visits after exclusion of patients < 18 years**	-47,529	173,679
**Number of ED visits after exclusion of referrals from triage to other care givers and exclusion of LWBS** ^**a**^	-16,577	157,102
**Number of ED visits after exclusion of visits with other complaints than dyspnoea** ^**b**^	-146,228	10,874
**Number of ED visits after exclusion of residents in other Swedish regions and in other countries at time of the visit**	-559	10,315

Cohort selection starting with all regional ED visits.

^a^LWBS: patients who left on their own accord without being seen by a doctor.

^b^I.e. not assigned “dyspnoea” according to the RETTS triage system [13].

### Ethical approval & reporting protocol

The study was approved by the Swedish Ethical Review Authority, no. 2021–02520. Informed consent was waived and the participants were instead given an opt-out possibility in accordance with the ethical approval. Information about the study and the opt-out possibility were published at Lund University Population Research Platform (LUPOP) and on the web page of Region Halland.

The study complies with the STROBE protocol.

### Labels

Our four labels AHF, eCOPD, pneumonia and "other diagnoses" were defined according to WHO diagnostic ICD-10 codes [14]. Heart failure was defined by ICD-10 codes I11.0, I13.0, I13.2 or I50, eCOPD by ICD-10 code J44 and pneumonia by ICD-10 codes J10.0, J11.0 or J12-J18. “Other diagnoses” was defined as all other ICD-10 codes. In in-hospital patients, the principal diagnosis in the discharge statement represented the label, while in non-admitted patients, the main ED discharge diagnosis made the label. A few patients had two registered main diagnoses in their EHR, these both were then used as labels. Thus, more than one label was allowed.

The nonspecific, symptom-based diagnosis “R06.0 dyspnoea” was common in patients discharged home from the ED. An adjudicating committee of three experienced emergency physicians manually reviewed all these patient visits (n = 1,070) to identify eventual missed AHF, eCOPD and pneumonia diagnoses by using all regional healthcare data up to 30 days after the ED visit. Two experts reviewed each visit until reaching an agreement. Diagnostic inaccuracy was then estimated as the number of missed diagnoses of AHF, eCOPD or pneumonia divided by the total number of visits with AHF, eCOPD or pneumonia, respectively.

Diagnostic inaccuracy in the study cohort was estimated as 4.5%, 6.6% and 1.9% in patients with AHF, eCOPD and pneumonia, respectively. After relabelling with the expert labels, 15.5% of visits had AHF, 14.0% had eCOPD, 13.3% had pneumonia, and 58.1% had other diagnoses, altogether slightly more than 100% due to multiple diagnoses. The label “other” represented, in addition to symptom-related diagnoses, numerous other diagnoses, of which pulmonary embolism, asthma and atrial fibrillation/flutter were the most common, but none was more prevalent in the cohort than 3.4% (S1 Table).

### Variables

For our deep learning model, all accessible, structured data produced in the patient´s trajectory through various care providers and IT systems in the entire region were collected by using the region´s comprehensive data analysis platform [15]. Data included electronic health records (EHRs) and administrative data from all primary care, outpatient specialist care, inpatient care, ambulance service and ED care. All private care within the region was also included except for a few minor private clinics which declined participation. The variables covered for example all diagnostic codes, procedure codes, prescribed and picked-up medications, vital signs, blood tests and referrals (Table 2). Free text in the medical charts, electrocardiograms (ECGs) and actual images were not accessed. To mirror the real-world setting, the data were not specifically selected or modified for the task. Blood samples were classified as low, normal, or high according to reference intervals. All data points were linked to patients and timestamps and then defined as "events".

**Table 2:** Clinical variables included.

Context	Source	Data
**1**	**Primary care**	Complaints Urgent/planned? Type of encounter (e.g., physical or digital) Care-provider category Procedures Primary and secondary diagnoses Referrals
**2**	**Outpatient specialist care**	Complaints Urgent/planned? Type of encounter (e.g., physical or digital) Organization/clinic Care-provider category Procedures Primary and secondary diagnoses Referrals
**3**	**Emergency department care**	Which hospital Ambulance/walk in Complaints Triage priority Care provider category Medications Procedures Primary and secondary diagnoses Hospital admittance or discharge Referrals
**4**	**Inpatient care**	Admitted from Urgent/planned? Organization/ward Medications Procedures Primary and secondary diagnoses Discharged to Referrals
**5**	**Ambulatory care**	Ambulance priority Medications Oxygen delivery Free airway? Semisitting position? Continuous positive airway pressure (CPAP)? Advance notice to ED? Pain Time: acknowledge of assignment, arrival to and departure from pick-up place, completion of assignment
**6**	**Others**	Ordinary medications, prescribed Ordinary medications, picked-up Number of picked up medication packages Distribution of medication to patient Blood samples and other laboratory tests Radiology exams, type of Smoking status
	**Self-derived and/or continuous numeric variables**	Age Sex Weight Number of earlier encounters: primary care, outpatient specialist care, ambulatory care, emergency department care, inpatient care Vital signs measured in primary care, outpatient specialist care, ambulatory care, ED care, in-hospital care: • Level of consciousness • Systolic and diastolic blood pressure • Pulse • Temperature • Oxygen saturation • Breathing frequency
	**Onsite variables at index visit**	Time at ED registration (hour, day, week) Which hospital Ambulance/walk in Number of concurrent ambulance assignments ED occupancy Triage priority Vital signs: • Level of consciousness • Systolic and diastolic blood pressure • Pulse • Temperature • Oxygen saturation • Breathing frequency

Included clinical variables and their different contexts and sources.

### Descriptive statistics

Percentage was calculated for categorical and ordinal data, while continuous variables were described by median and IQR by using SPSS version 29.0 [16].

### Model design

We designed a *clinical attention-based recurrent encoder network* (CareNet) to accurately diagnose ED patients based on their triage variables and clinical history (Table 2). CareNet draws inspiration from advancements in natural language processing (NLP) for document classification tasks [17]. We adopted a similar approach to represent the patient’s health status at the index time.

**Fig 1:**
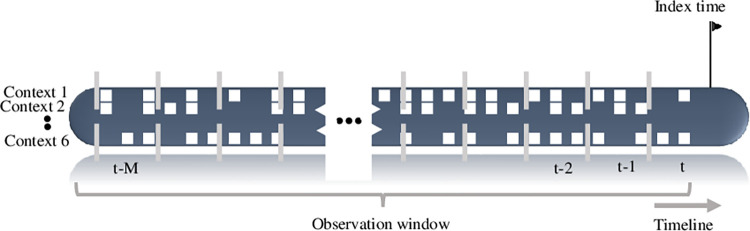
An ED patient visit. A single ED patient visit in the health care system. The medical past is divided into periods and clinical contexts.

We began by equally segmenting a patient’s timeline into *M* periods (Fig 1). Next, we looked for clinical events in each period.

**Fig 2:**
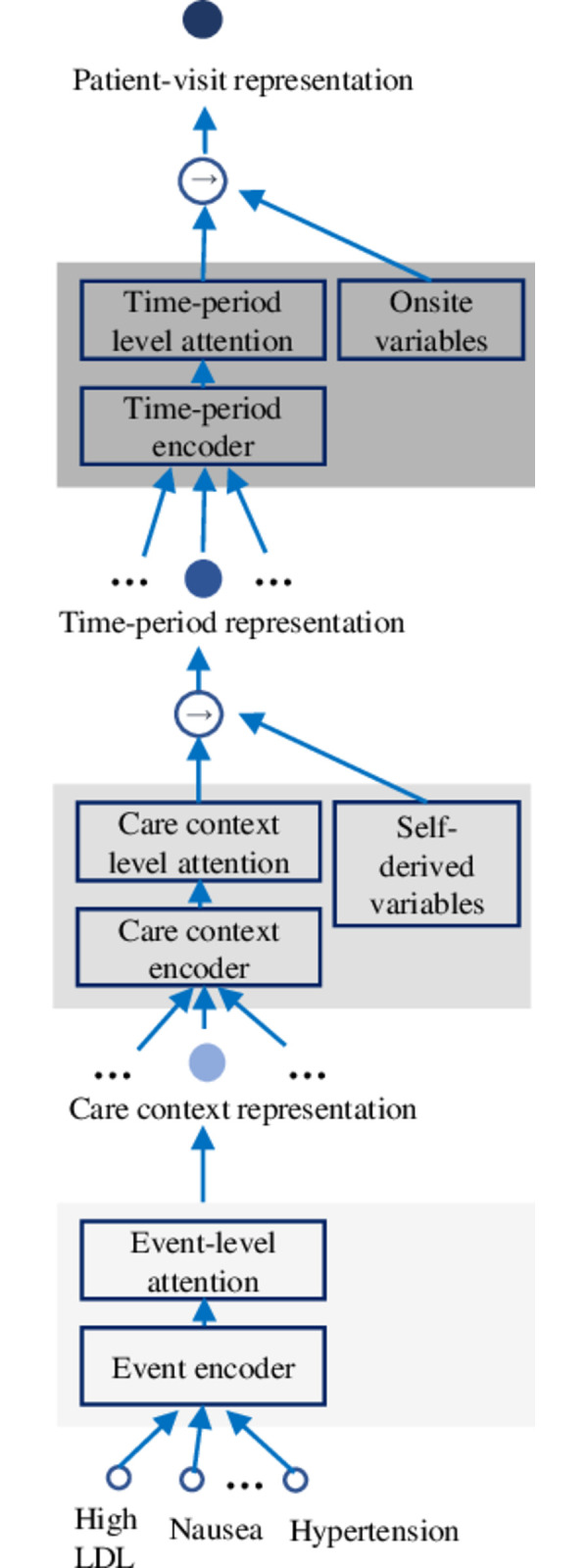
Hierarchical attention network of a patent visit. The hierarchical attention network of CareNet analysing a patient visit. Circles represent vectors.

CareNet’s three-layer hierarchical structure is designed to mimic a clinician’s approach to capture time and context awareness for each clinical event. Fig 2 illustrates the CareNet architecture. Each layer has two key components. The encoder has bidirectional gated recurrent units (GRUs) [18]. It summarizes both directions of the input to incorporate neighbourhood information into each input embedding. Since not all inputs contribute equally to the higher-level representation, we have an attention block that performs a weighted aggregation of the input embeddings by learning how much an input embedding should contribute to the higher-level representation. In this text, the terms *embeddings* and *representations* are used interchangeably to refer to numerical vectors representing individual clinical events, care contexts, or care time segments.

Given events in a specific care context and period, the event encoder converts these events to numeric embeddings, gleaned via pretrained skip-gram initialization [19] and encodes the other events (neighbourhood) information into each embedding. The event-level attention block calculates the importance of each event embedding to generate the care context embedding (Fig 2). The process is then repeated in the middle, context layer. The care context encoder encodes the other context neighbourhood information into each context embedding. The care context attention block then calculates how much each context contributes towards the top, time-period, and layer and accordingly aggregates the context embeddings. Here, we concatenate the output of the context attention block with self-derived or continuous numeric variables (Table 2) to obtain the time-period representation. The final layer, the time-period encoder, encodes all time information into each time-period embedding. This is followed by a time-period level attention block that calculates how much different periods contribute to the patient representation and aggregates the time-period embeddings accordingly. The output of the time-period attention block is concatenated with onsite variables (Table 2) to obtain the final patient visit representation. This final patient visit representation is then passed through a feed-forwards neural network to obtain the diagnosis label distribution. Evidential loss is calculated using labels. CareNet is trained in an end-to-end manner by minimizing the loss using backpropagation. A detailed description of patient visit representation learning and the training process has been added to the supplement (see S1 Text).

### Experiments and evaluation

Multiple experiments were conducted, including adjustment of the observation window from five to one year and using both raw and expert-derived labels. For all the experiments, we conducted 10-fold cross-validation and, within each fold, performed 10 bootstrapped evaluations using 90% of the evaluation set. This approach resulted in a final 10x10 matrix of area under the receiver operating characteristic curve (AUROC) values, providing a comprehensive performance assessment that reflects both cross-validation and bootstrapping techniques. We reported the median micro AUROC (2.5–97.5 percentile) on the evaluation fold, i.e., each patient visit was given the same weight. The multilabel model design enabled a probability between 0 and 1 for each label (diagnose), i.e., the sum of the label probabilities might be more than 100%.

We analysed attention behaviours over the cohort to explore an average pattern and how clinical events in the different contexts contribute to the classification. Since there are no commonly implemented diagnostic scores for dyspnoeic ED patients, we created a baseline model for comparison. We built a tree-based CatBoost model [20] using expert-derived clinical features based on literature studies [21–24] and clinical reasoning among the researchers. The nine variables were age, sex, a diagnosis of heart failure, COPD or pneumonia registered anywhere in the regional health care system within five years prior to index visit, a diagnosis of AHF, eCOPD or pneumonia registered at one of the regional EDs within one year prior to index visit and temperature, measured in index visit triage. The labels were defined with the same ICD-10 codes as the CareNet model.

For the CatBoost model, we leveraged the MissForest algorithm that iteratively builds random forest models to impute missing instances of input features [25]. The CatBoost variables had no missing data, besides temperature with 2.1% missing values. For CareNet, we fed raw data with "NA" markers for missing instances, allowing the model to autonomously deduce missing values as part of its training.

AUROC sensitivity and specificity for each label were reported as medians (2.5–97.5 percentile), calculated by using 10x10 matrices of AUROC values after cross-validation and bootstrapping techniques. Sensitivity and specificity were defined as maximum sensitivity with a specificity above 75.0% and its corresponding specificity.

We analysed CareNet attention behaviours to explore how clinical events contribute to the classification. To estimate the diagnostic importance for each type of event for the whole cohort, we multiplied each patient visit´s event weight by the weight of the event´s context and the weight of the event´s period. Weights of the same type of events from different patients were added to make a list of diagnostic variables in order of diagnostic ability.

## Results

### Descriptive statistics

Among the 10,315 visits, the number of unique patients was 6,967. In the expert-labelled cohort, 15.5% of visits had AHF, 14.0% had eCOPD and 13.3% had pneumonia. 97 patient visits (0.9% of the cohort) had two of AHF, eCOPD or pneumonia as main diagnosis, and thus two labels. The median number of unique prior events per ED visit was 1,095 (interquartile range, IQR 459–2,310) with five years of data and 352 events (IQR 134–838) with one year of data. The median age among the visits was 75 years (IQR 62–84), and women constituted slightly more than half of the cohort (Table 3).

**Table 3:** Cohort characteristics.

	All cohort	AHF^a^	eCOPD^b^	Pneumonia^c^	Other diagnoses^d^
***Visits*, *N (%)***	10,315 (100.0)	1,596 (15.5)	1,445 (14.0)	1,376 (13.3)	5,995 (58.1)
***Unique patients*, *N***	6,967	1,202	768	1,219	4,794
**Age, *median (IQR)***	75 (62–84)	83 (77–89)	76 (69–82)	77 (66–86)	71 (53–81)
***Sex*, *N (%)***					
**Male**	4,928 (47.8)	860 (53.9)	632 (43.7)	676 (49.1)	2,804 (46.8)
**Female**	5,387 (52.2)	736 (46.1)	813 (56.3)	700 (50.9)	3,191 (53.2)
***Medical history***					
***Charlson Comorbidity Index*, *median (IQR)***	1.0 (0.0–2.0)	2.0 (0.0–3.0)	1.0 (1.0–2.0)	0.0 (0.0–2.0)	0.0 (0.0–2.0)
***Heart failure diagnosis*, *previous year*** ^***e***^ **, *N (%)***	2,298 (22.3)	870 (54.5)	410 (28.4)	234 (17.0)	815 (13.6)
***COPD diagnosis*, *previous year*** ^***e***^ **, *N (%)***	2,322 (22.5)	260 (16.3)	1,181 (81.7)	286 (20.8)	655 (10.9)
***Pneumonia diagnosis*, *previous month*** ^***e***^ **, *N (%)***	425 (4.1)	35 (2.2)	47 (3.3)	151 (11.0)	197 (3.3)
***No*. *of primary care encounters previous year*** ^***f***^ **, *median (IQR)***	11.0 (5.0–22.0)	17.0 (9.0–29.0)	13.0 (6.0–24.0)	10.0 (5.0–19.8)	10.0 (4.0–20.0)
***No*. *of outpatient specialist encounters previous year*** ^***f***^ **, *median (IQR)***	3.0 (0.0–9.0)	4.0 (1.0–10.0)	3.0 (1.0–9.0)	2.0 (0.0–8.0)	3.0 (0.0–8.0)
***No*. *of emergency department visits previous year*, *median (IQR)***	1.0 (0.0–3.0)	1.0 (0.0–3.0)	2.0 (0.0–4.0)	1.0 (0.0–2.0)	1.0 (0.0–2.0)
***No*. *of in-hospital visits last year*, *median (IQR)***	1.0 (0.0–2.0)	1.0 (0.0–3.0)	1.0 (0.0–3.0)	1.0 (0.0–2.0)	0.0 (0.0–2.0)
***Index visit***					
***Hospital N (%)*** ^***g***^					
**Hospital 1**	5,405 (52.4)	906 (56.8)	804 (55.6)	633 (46.2)	3,114 (52.1)
**Hospital 2**	4,887 (47.4)	689 (43.2)	639 (44.2)	738 (53.8)	2,865 (47.9)
***Arrival time to emergency department N (%)***					
**Monday-Friday, 8:00 am-8:59 pm**	6,278 (60.9)	1,068 (66.9)	810 (56.1)	806 (58.6)	3,667 (61.2)
**Saturday-Sunday, 8:00 am-8:59 pm**	1,934 (18.7)	298 (18.7)	275 (19.0)	282 (20.5)	1,095 (18.3)
**Nighttime, 9:00 pm-7:59 am**	2,103 (20.4)	230 (14.4)	360 (24.9)	288 (20.9)	1,233 (20.6)
***Ambulance arrival*, *n (%)***	5,123 (49.7)	890 (55.8)	1,010 (69.9)	830 (60.3)	2,445 (40.8)
***Triage priority*, *n (%)***					
**Priority 1**	596 (5.8)	99 (6.2)	104 (7.2)	139 (10.1)	263 (4.4)
**Priority 2**	5,028 (48.7)	902 (56.5)	937 (64.8)	801 (58.2)	2,458 (41.0)
**Priority 3**	3,960 (38.4)	578 (36.2)	385 (26.6)	398 (28.9)	2,617 (43.7)
**Priority 4**	677 (6.6)	14 (0.9)	16 (1.1)	36 (2.6)	611 (10.2)
**Priority 5**	54 (0.5)	3 (0.2)	3 (0.2)	2 (0.1)	46 (0.8)

Cohort characteristics of visits labelled AHF, eCOPD, pneumonia and “other diagnoses”, considering the adjudicating committee´s result.

^a^Heart failure: ICD-10 code I11.0, I13.0, I13.2 or I50.

^b^Chronic obstructive pulmonary disease (COPD): ICD-10 code J44.

^c^Pneumonia: ICD-10 code J10.0, J11.0 or J12-J18.

^d^Other diagnoses: all other ICD-10 codes.

^e^Registered anywhere in the regional health care system.

^f^Encounters included visits, digital meetings and phone calls.

^g^Missing value in all cohort: n = 23 (0.2%).

### Diagnostic performance

CareNet´s performance, measured by median micro AUROC (2.5–97.5 percentile) was 87.0% (84.8–88.3%), by using one year of data and expert labels (Table 4).

**Table 4:** Diagnostic performance.

Model	Median micro AUROC (%, 2.5–97.5 percentile)
**CareNet: With one year of data, with expert labels**	87.0 (84.8–88.3)
**CareNet: With five years of data, with expert labels**	86.9 (82.2–88.6)
**CareNet: With one year of data, without expert labels**	87.0 (85.0–88.8)
**CatBoost: With five years of data, without expert labels (baseline)**	81.4 (77.5–86.6)

Diagnostic performance for the CareNet model using one versus five years of data prior to index visit and with and without expert labels compared to the baseline CatBoost model.

CareNet performed substantially better than the CatBoost baseline model, which had a median performance of 81.4% (77.5–86.6%) (Table 4). Feeding the CareNet model with five years of data, compared to one year of data, did not improve performance. We also compared performance with and without expert labels, and the performance remained the same (Table 4).

CareNet AUROC was considerably higher for patients ≤ 75 years compared to older patients (91.5% versus 81.2%) and for patients without all three diagnoses of heart failure, COPD and pneumonia recorded in their medical history in the previous year compared to patients with all three diagnoses registered (88.0% versus 69.0%) (S2 Table).

**Fig 3:**
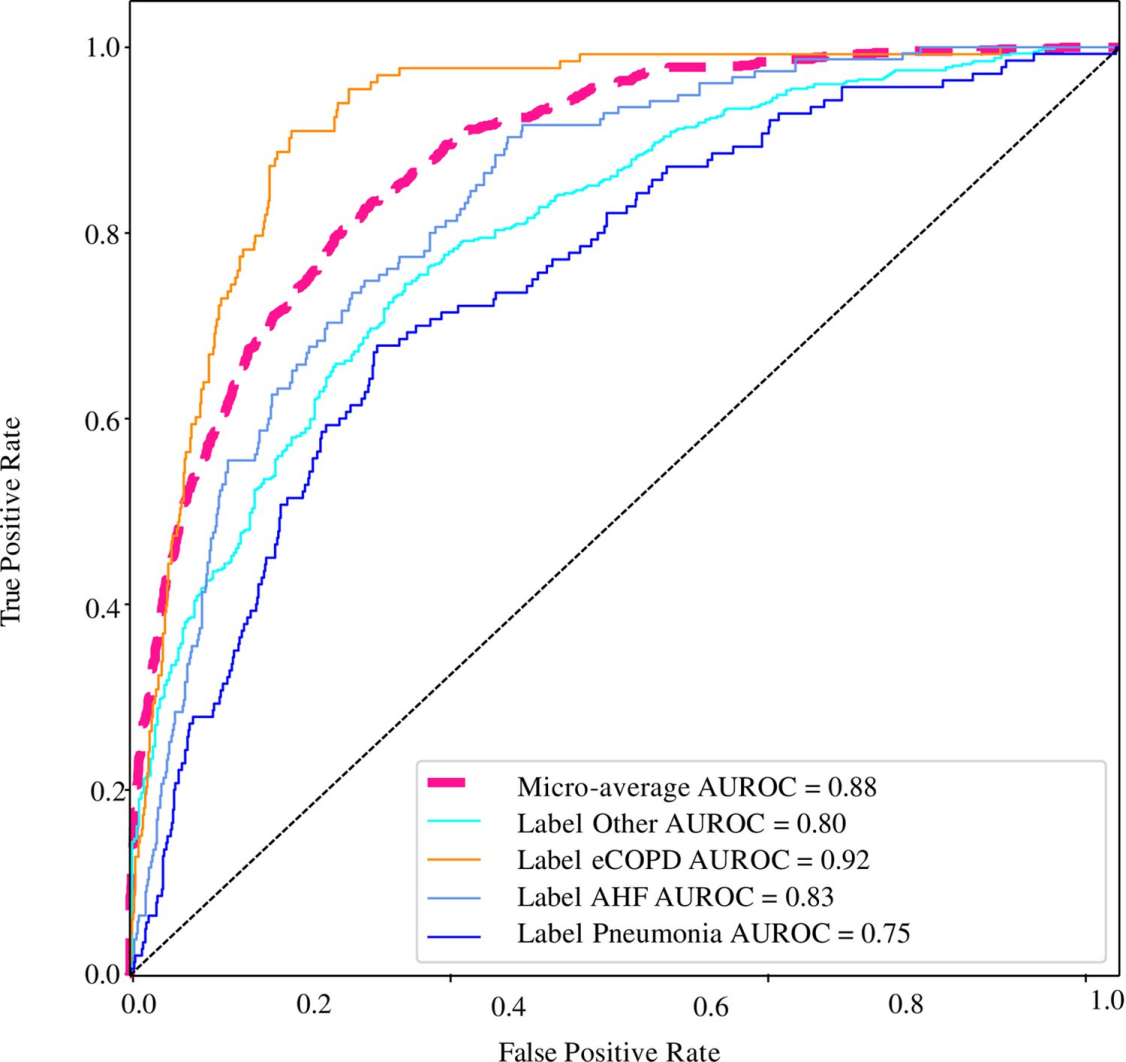
CareNet performance across different diagnostic labels. CareNet AUROC using one year of data prior to index visit and expert labels. An illustrative example from one of the validation folds with the highest micro AUROC.

CareNet AUROC for each diagnosis is shown in an illustrative example from one of the validation folds (Fig 3).

CareNet median sensitivity for AHF, eCOPD, pneumonia and the “other” label was 74.5%, 92.6%, 54.1% and 64.2%, respectively, with a specificity set above 75.0% (Table 5). This result was overall slightly inferior to that of the CatBoost baseline model; AHF 71.3%, eCOPD 93.0%, pneumonia 59.3% and “other” diagnoses 67.3% with a specificity set above 75.0% (Table 5).

**Table 5:** CareNet sensitivity and specificity.

		AHF	eCOPD,	Pneumonia	Other
**CareNet**	**Median sensitivity (%, 2.5–97.5 percentile)**	74.5 (65.0–82.0)	92.6 (84.5–97.7)	54.1 (39.9–70.9)	64.2 (55.3–72.6)
	**Median specificity (%, 2.5–97.5 percentile)**	75.5 (75.0–76.6)	75.3 (75.0–76.0)	75.8 (75.0–77.4)	77.4 (75.1–83.6)
**CatBoost baseline model**	**Median sensitivity (%, 2.5–97.5 percentile)**	71.3 (59.8–81.7)	93.0 (85.1–98.5)	59.3 (43.5–71.2)	67.3 (58.6–72.1)
	**Median specificity (%, 2.5–97.5 percentile)**	78.4 (75.2–83.5)	81.6 (78.5–85.8)	78.1 (75.2–83.4)	76.6 (75.0–80.3)

CareNet sensitivity and specificity compared to the CatBoost model.

### Variable weight

In the CareNet design, the weights of all patients´ clinical events, multiplied by the importance of their clinical context and time period, were added to a list of a total of 1,596 variables in order of diagnostic ability over the whole cohort. The top 30 of those are shown in Table 6. The model gave most attention to prior diagnoses of heart failure or COPD, followed by daily smoking, atrial fibrillation/flutter, life management difficulties and maternity care.

**Table 6:** CareNet top 30 ranked variables.

	Variable
**1**	Heart failure, primary diagnosis
**2**	Chronic obstructive pulmonary disease (COPD), primary diagnosis
**3**	Chronic obstructive pulmonary disease (COPD), secondary diagnosis
**4**	Daily smoking
**5**	Atrial fibrillation/flutter, ED complaint
**6**	Life management difficulties, primary diagnosis
**7**	Appointment for health talk, maternal health services
**8**	Bladder cancer, specialist care complaint
**9**	ICD (implantable cardioverter defibrillator), specialist care complaint
**10**	Contact lens, primary care complaint
**11**	First appointment, maternal health services
**12**	Medication against obstructive airways, collected recipe
**13**	Head trauma, ED complaint
**14**	Examination and observation for other specified reasons, primary diagnosis
**15**	Chronic obstructive pulmonary disease (COPD), primary care complaint
**16**	Investigation, primary care complaint
**17**	Nose bleeding, ED complaint
**18**	Prostate, specialist care complaint
**19**	Endocrine, specialist care complaint
**20**	Diabetes, specialist care complaint
**21**	Kidney failure, primary diagnosis
**22**	Wound, primary care complaint
**23**	Other pulmonary heart diseases, primary diagnosis
**24**	Cataract, specialist care complaint
**25**	Other bacterial intestinal infections, primary diagnosis
**26**	Hernia, complaint specialist care
**27**	Endocrinological clinic, organizational code
**28**	Evaluation of need or function, measurement code
**29**	Neurological deficit, ED complaint
**30**	Pneumonia, organism unspecified, primary diagnosis

CareNet top 30 ranked variables by using data up to one year prior to index visit as well as expert labels.

### Interpretable diagnostics

**Fig 4:**
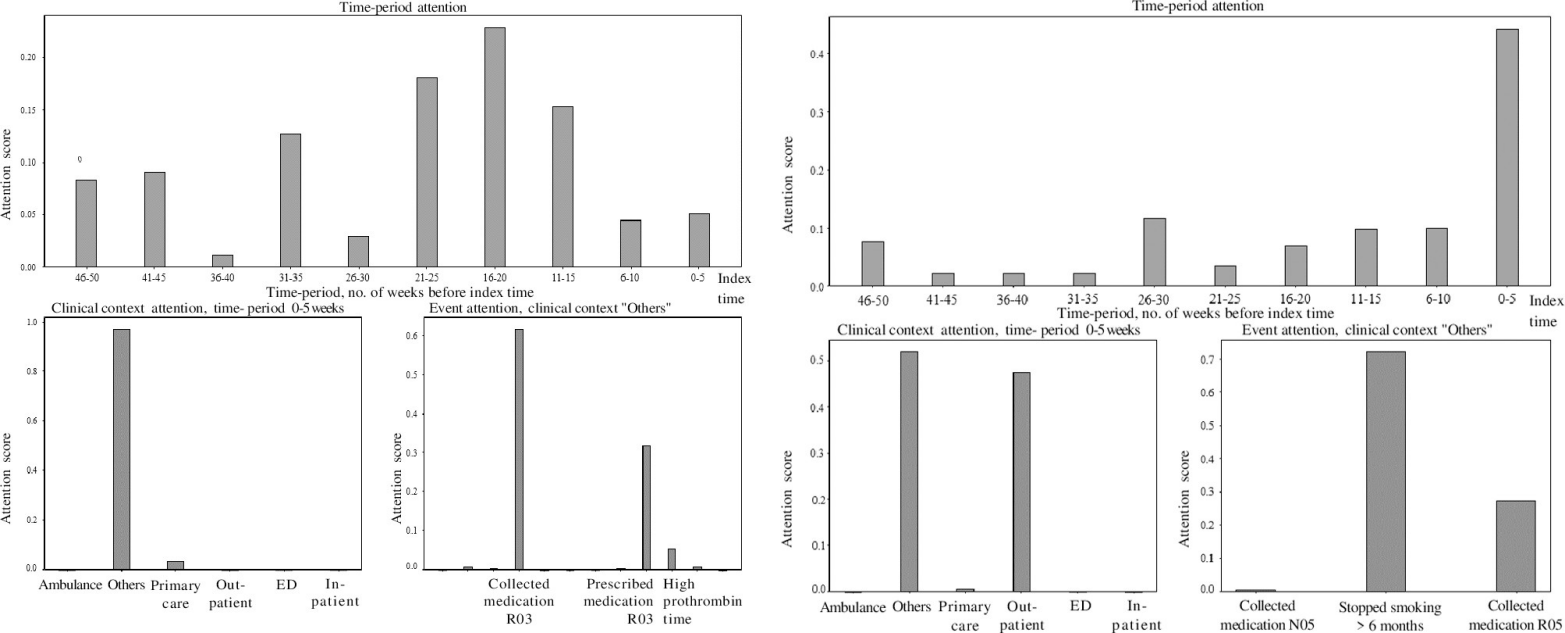
A-B. Individual patient attention plots with diagnosis AHF and pneumonia. 4A. Attention plot for a patient with AHF. The period 16–20 weeks prior to the index visit has the highest attention, i.e., is most important for diagnosis (diagram on top). During this most important period, the “others” category (diagram down to the left) is most important for diagnosis. The “others” variables are described in Table 2. Among the “others” variables, attention is highest for collected and, after that, prescribed medication of “R03 drugs for obstructive airway diseases” (diagram down to the right). 4B. Attention plot for a patient with pneumonia. The period 0–5 weeks prior to the index visit has the highest attention, i.e., is most important for diagnosis (diagram on top). During this most important period, the “others” category (diagram down to the left) is most important for diagnosis. The “others” variables are described in Table 2. Among the “others” variables, attention is highest for “stopped smoking > 6 months ago” and “collected medication R05 cough and cold preparations” (diagram down to the right).

Each individual patient visit received their own unique attention plot based on individually selected variables (see examples in Fig 4A, 4B). Seen on top in each figure, starting from index time, each prior five-week period is weighted according to its diagnostic importance. The most important period displays its six clinical contexts down to the left. The most important clinical context shows its most substantial clinical events down to the right.

## Discussion

In this population-wide cross-sectional study we designed an AI-based diagnostic support for dyspnoea at the time of ED triage by analysing unselected real-world data from a complete regional health care system. A novel deep learning model, CareNet, was designed, which achieved a median AUROC of 87.0% in discriminating patients with AHF, eCOPD, pneumonia and other conditions already in the very beginning of the ED visit, before any blood tests, imaging or physician assessment is being performed. 1,596 variables were also ranked by their diagnostic abilities, for further exploration.

We exposed a broad, unselected dataset to a complex deep learning model to open-endedly explore if there are predictors that the medical field has not assessed before. This is important since today´s diagnosis of dyspnoea in emergency care is surprisingly erroneous [8, 11, 26], with one-third, up to almost half, of admitted elderly patients receiving improper treatment for their diagnoses of AHF, eCOPD or pneumonia in the ED [10]. An other reason was to enable individualized medicine in which the variables are not defined beforehand but rather selected by the model for each individual patient.

The CareNet AUROC was considerably higher for younger patients and substantially lower for patients with all three previous diagnoses of heart failure, COPD and pneumonia (S2 Table). This seems compatible with a study performed on diagnostic performance in emergency care at hospitals in the United States [10]. However, the difference in performance between women and men was small (S2 Table).

When the model was fed five years of data instead of one year of data, the performance remained the same (Table 4). The algorithm might have obtained too wide data when including five years, related to the sample size, or perhaps much of the information, for example diagnoses, was repeated in the older data. A third explanation might be that the model prioritizes the most recent time periods in the model.

CareNet micro AUROC performance was considerably higher (87.0%) than that of our CatBoost model (81.4%). We prefer to use micro-averaging rather than macro-average since the classes are unbalanced, however micro-averaging needs the individual models to be calibrated. The CatBoost models for each class may be less calibrated compared to the single CareNet model, explaining the lower average micro AUROC value for the CatBoost model. When comparing sensitivity at specificity higher than 75.0%, the CatBoost model appeared slightly better overall (Table 5). A disadvantage for the deep learning model might be that there were rather few, strong predictors in the prediction task which do not reward a complex model or that CareNet´s large dataset held too much noise.

The CareNet performance might be compared with a German study where emergency medicine-trained anaesthesiologists in the ambulance diagnosed the patients immediately after initial triage [8]. Although the cohort was slightly different than ours, the diagnostic accuracies for AHF, eCOPD and pneumonia were 77.4%, 82.6% and 49.3%, respectively. These results suggest that it is easiest to diagnose eCOPD and hardest to diagnose pneumonia, which is similar to the CareNet performance (Table 5).

For an open-ended search of predictors, we presented an AI-generated list of 1,596 unselected variables by order of diagnostic ability, to enable further testing and evaluation in a simpler model. In the list, the highest weighted variables seem medically reasonable: prior diagnoses of heart failure and COPD diagnosis, daily smoking, atrial fibrillation/flutter, life difficulties and maternal care (Table 6).

An important aim of our model was interpretability. Generally, interpretability refers to the extent of a human’s ability to understand and reason about a model [27], a field which is believed to be important but underexplored [28]. Therefore, CareNet analyses and presents clinical events placed in time and clinical context for each patient, as we believe clinicians intuitively do, e.g., a blood test taken a month ago at an earlier ED visit. (Fig 4A, 4B). In an imagined future, all graphic bars might be “clickable,” displaying the individual patient´s unique underlying diagnostic factors to the clinician.

### Strengths and limitations

As a strength, this research was based on a complete regional population and entire regional health care system data, including all regional emergency care.

We used all accessible regional health care system data, without further selection or modification, which reduces selection bias. It also makes the model more generic for other research questions. Additionally, real-world data mimic the actual clinical situation. As a limitation, our data only included structured data and not free text, images, or ECGs. Also, the model does not compensate for bias in care consumption or variability in clinician behaviour.

A strength of this study is that we included both admitted and discharged ED patients. Many studies include only admitted patients, even though the disposition decision is made later. The reason is inaccuracy of diagnostic outcome labels in EHR data, a well-known limitation especially in patients discharged from the ED to home, when you cannot rely on an in-hospital discharge summary, often summarizing several days of further testing and evaluation [29]. To overcome this, experienced emergency physicians manually reviewed more than one thousand patient visits. We believe the magnitude of the diagnostic inaccuracy was acceptable for our research question, and the performance did not differ when comparing data with and without expert labels (Table 4). Nevertheless, diagnostic uncertainty must be considered and controlled in future studies and implementations.

### Future implications

Short-term, AI might be focused on methodological development rather than implementation, suggested by researchers [30]. Future studies could further explore our AI-derived list of 1,596 weighted variables. A suggestion would be to carefully select variables only from the upper part of the list, and test them in a smaller, suitable model, aiming for high performance.

Images, ECGs and unstructured data might be added to the CareNet model, which allows multi-modal analyses, to evaluate the effect on performance.

Only the main diagnosis registered in the EHR has been used as label in this study. This reflects the most important diagnosis to identify and treat during the ED visit. A following study might also include secondary diagnoses in the EHR, to mirror possible additional conditions which might worsen the main diagnosis, and maybe also need treatment.

In a future study, the model could be trained and validated in a primary care setting, using index visit variables originating from primary care rather than emergency care. This would enable the provision of diagnostic support to primary care physicians as well.

According to earlier research [31], a complex model might be more robust among specific patient subgroups. We may compare performance of our model with corresponding baseline performance in selected patient subgroups.

## Conclusion

We developed an AI tool for diagnosing dyspnoeic adults at the time of triage in the ED by analysing comprehensive data from an entire regional healthcare system. The AI is interpretable for clinicians, as it contextualises data within its clinical setting and timeframe. Today, we generate new, machine-derived insights into previously unknown but significant diagnostic predictors. Looking ahead, we foresee a future of more individualised medicine.

## Supporting information

S1 Table The five most prevalent diagnoses. Prevalence, in the total study cohort, of the five most common diagnosis codes within the “other” label group.
(DOCX)

S2 Table CareNet performance in subgroups. Comparison of CareNet´s diagnostic performance in different cohort subgroups. All models include one year of data prior to index visit and expert labels.
(DOCX)

S1 Text CareNet mathematical background. Mathematical background of the Clinical attention-based recurrent encoder network (CareNet) design.
(DOCX)
