# Supplementary material for: A novel interpretable deep learning model for diagnosis in emergency department dyspnoea patients based on complete data from an entire health care system
Source: PLoS One. 2024 Dec 27;19(12):e0311081. doi: 10.1371/journal.pone.0311081 (PMC11676563; doi:10.1371/journal.pone.0311081)
Supplement: S1 Table — Prevalence, in the total study cohort, of the five most common diagnosis codes within the “other” label group. (DOCX) [file pone.0311081.s001.docx]

**S1 Table.** **The five most prevalent diagnoses.**

|  | **ICD-10 code and name** | **Prevalence (%)** |
| --- | --- | --- |
| **1.** | R06 Abnormalities of breathing | 10.7 |
| **2.** | R07 Pain in throat and chest | 4.3 |
| **3.** | I26 Pulmonary embolism | 3.4 |
| **4.** | J45 Asthma | 2.3 |
| **5.** | I48 Atrial fibrillation and flutter | 1.9 |

Prevalence, in the total study cohort, of the five most common diagnosis codes within the “other” label group.
