# Supplementary material for: A novel interpretable deep learning model for diagnosis in emergency department dyspnoea patients based on complete data from an entire health care system
Source: PLoS One. 2024 Dec 27;19(12):e0311081. doi: 10.1371/journal.pone.0311081 (PMC11676563; doi:10.1371/journal.pone.0311081)
Supplement: S2 Table — Comparison of CareNet´s diagnostic performance in different cohort subgroups. All models include one year of data prior to index visit and expert labels. (DOCX) [file pone.0311081.s002.docx]

**S2 Table. CareNet performance in subgroups.**

|  | **Median micro AUROC (%, 2.5–97.5 percentile)** |
| --- | --- |
| **All cohort** | 87.0 (84.8–88.3) |
| **Females** | 87.3 (83.9–90.4) |
| **Males** | 86.2 (84.2–88.9) |
| **0-2 diagnoses in medical history^a^** | 88.0 (85.8–89.3) |
| **3 diagnoses in medical history^a^** | 69.0 (54.9–80.8) |
| **Age <75 years** | 91.5 (89.6–93.3) |
| **Age >75 years** | 81.2 (77.3–83.1) |

Comparison of CareNet´s diagnostic performance in different cohort subgroups. All models include one year of data prior to index visit and expert labels.

^a^Visits having none, one, two or all three diagnoses of heart failure, chronic obstructive pulmonary disease (COPD) and pneumonia registered anywhere in the regional electronic health care system in the preceding 12 months. The diagnoses were defined with the same ICD-10 codes as in the CareNet model.
