# Supplementary material for: A novel interpretable deep learning model for diagnosis in emergency department dyspnoea patients based on complete data from an entire health care system
Source: PLoS One. 2024 Dec 27;19(12):e0311081. doi: 10.1371/journal.pone.0311081 (PMC11676563; doi:10.1371/journal.pone.0311081)
Supplement: S1 Text — Mathematical background of the Clinical attention-based recurrent encoder network (CareNet) design. (DOCX) [file pone.0311081.s003.docx]

**S1 Text.** **CareNet mathematical background.** Mathematical background of the Clinical attention-based recurrent encoder network (CareNet) design.

**Problem setup**

Figure 1 in the manuscript illustrates our problem setup as a single-patient visit's care trajectory in an EHR, which is composed of different patient-specific details reported at different time periods and contexts within the healthcare system. For instance, a diagnosis assigned to a patient has a period and a context (primary care, outpatient specialist care, ambulatory care, emergency department care, inpatient care) associated with it, both having important clinical value. The goal is to capture all clinical information with periods and contexts up to the index time and numerically represent it (i.e., the health state of the patient at index time), enabling its classification into different disease groups. A detailed list of data types derived from different care contexts has been added to Table 2 in the manuscript.

We approach the problem of representing the health state of the patient at the index time using natural language processing (NLP)-inspired attention modules ^17^. For instance, we begin by segmenting the patient visit trajectory into equal periods (or paragraphs). Within each period, we have six different contexts (or sentences) derived from different healthcare contexts. Each context constitutes clinical events (or words) recorded in the specific healthcare context and period. Overall, the ordered sequence of time-periods (or paragraphs) makes up the patient-visit's health state (or a document).

**Generating patient visit representation**

In the context of NLP, we developed a three-layer hierarchical document (patient) embedding module consisting of word-level (clinical events), sentence-level (healthcare contexts) and paragraph-level (periods) embeddings and attention modules.

Let us consider a single patient visit $p$ with $M$ periods, where each period consists of $K$ contexts (six in our case) and each context consists of $N$ clinical events. Vectors and matrices are represented in lower and upper case bold, respectively. $\boldsymbol{p}$ is the vector representation of patient visit $p.\boldsymbol{t}_{m}$ is the vector representation of period $m$ of patient visit $p.\boldsymbol{s}_{mk}$ is the vector representation of context $k$ within period $m$ of patient visit $p.\boldsymbol{c}_{mkn}$ is the $n^{\text{th }}$ code vector from context $k$ in period $m$ of patient visit $p$. For simplicity, we ignore the suffixes hereafter.

For word (or event) level embedding and attention, we first embed all the $N$ clinical events from a care context $s$ and period $m$ of patient visit $p$ into a vector space via a pretrained skip-gram initialization ^18^. These event vectors $\boldsymbol{c}$ are then passed through bidirectional gated recurrent units (GRUs) to obtain an intermediate representation $\boldsymbol{h}^{\boldsymbol{c}}$ for each event, such that it also incorporates the contextual information of other events in $s$. Since not every event contributes equally to the overall context representation, we apply Bahdanau attention using a single-layer NN with weights $\boldsymbol{W}^{c}$ and bias $\boldsymbol{b}^{c}$ as follows to obtain event-level attention $\boldsymbol{a}_{\boldsymbol{c}}$ and context representation $\boldsymbol{s}$:

$\boldsymbol{u}_{\boldsymbol{c}}=\tanh\left( \boldsymbol{W}^{c}\boldsymbol{h}^{c}+\boldsymbol{b}^{c} \right);\boldsymbol{a}_{\boldsymbol{c}}=\frac{\exp\left( \boldsymbol{u}_{c}^{T}\boldsymbol{u}^{c^{'}} \right)}{\sum_{n=1}^{N} \exp\left( \boldsymbol{u}_{\boldsymbol{c}}^{T}\boldsymbol{u}^{c^{'}} \right)};\boldsymbol{s}=\sum_{n=1}^{N} \boldsymbol{a}_{c}\boldsymbol{h}^{c}$ (1)

where $\boldsymbol{u}^{c^{'}}$ is the event-level self-attention vector, which is randomly initialized and jointly learned during training.

For context-level attention, we follow similar steps. We stack another bidirectional GRU and a single-layer attention NN to generate care context-level attention $\boldsymbol{a}_{\boldsymbol{s}}$ and time-period representation $\boldsymbol{t}$ as follows:

$\boldsymbol{u}_{\boldsymbol{s}}=tanh\left( \boldsymbol{W}^{s}\boldsymbol{h}^{s}+\boldsymbol{b}^{s} \right);\boldsymbol{a}_{s}=\frac{exp\left( \boldsymbol{u}_{s}^{T}\boldsymbol{u}^{s^{'}} \right)}{\sum_{k=1}^{K} exp\left( \boldsymbol{u}_{s}^{T}\boldsymbol{u}^{s^{'}} \right)};\boldsymbol{t}^{'}=\sum_{k=1}^{K} \boldsymbol{a}_{s}\boldsymbol{h}^{s}$ (2)

where $\boldsymbol{h}^{s}$ is the intermediate context representation (after passing $\boldsymbol{s}$ through a bidirectional GRU), $\boldsymbol{u}^{\boldsymbol{s}^{'}}$ is the context-level self-attention vector, and $\boldsymbol{W}^{s}$ and $\boldsymbol{b}^{s}$ are context-level NN weights and bias, respectively. The time-period representation $\boldsymbol{t}$ is finally generated by concatenating $\boldsymbol{t}^{'}$ with other continuous value features such as age and vital signs during that time-period.

Next, for time-period level attention, we repeat the process. We stack another bidirectional GRU and a single-layer attention NN to generate time-level attention $\boldsymbol{a}_{\boldsymbol{t}}$ and patient visit representation $\boldsymbol{p}$ as follows:

$\boldsymbol{u}_{\boldsymbol{t}}=tanh\left( \boldsymbol{W}^{t}\boldsymbol{h}^{t}+\boldsymbol{b}^{t} \right);\boldsymbol{a}_{\boldsymbol{t}}=\frac{exp\left( \boldsymbol{u}_{\boldsymbol{t}}^{T}\boldsymbol{u}^{t^{'}} \right)}{\sum_{m=1}^{M} exp\left( \boldsymbol{u}_{\boldsymbol{t}}^{T}\boldsymbol{u}^{t^{'}} \right)};\boldsymbol{p}^{'}=\sum_{m=1}^{M} \boldsymbol{a}_{t}\boldsymbol{h}^{t}$ (3)

where $\boldsymbol{h}^{t}$ is the intermediate time-period and context representation (after passing $\boldsymbol{t}$ through a bidirectional GRU), $\boldsymbol{u}^{t^{'}}$ is the time-period level self-attention vector, and $\boldsymbol{W}^{t}$ and $\boldsymbol{b}^{t}$ are time-level NN weights and bias, respectively.

The patient visit representation $\boldsymbol{p}$ is finally generated by concatenating $\boldsymbol{p}^{'}$ with the index visit's onsite triage variables, such as temperature and pulse.

**Loss computation and training**

Given our patient visit representation $\boldsymbol{p}$, we finally map it to a 4-node output layer. We then calculate the multilabel evidential loss proposed in ^32^ using ground truth labels for training all the NN weights.

**Experiments and evaluation**

We conducted experiments using varying amounts of input data. This involved adjusting the observation window (five years and one year) before the index time and utilizing both raw EHR labels and expert-verified variables. In each experiment, we employed 10-fold cross-validation with minimal hyperparameter tuning. Of note, we are aware of the potential minor impact stemming from the absence of a separate hold-out set, which could influence the reported results. Further validation through external datasets could enhance the robustness of our findings.

Within the context of these experiments, our methodology involved performing 10 bootstrapped evaluations in each fold using 90% of the evaluation set. This approach resulted in a final 10x10 matrix of AUROC values, providing a comprehensive performance assessment that considers both cross-validation and bootstrapping techniques, reported as median micro AUROC (2.5–97.5 percentile). AUROC sensitivity and specificity for each label were also calculated by using 10x10 matrices of AUROC values after cross-validation and bootstrapping techniques. Sensitivity and specificity were defined as maximum sensitivity with a specificity above 75% and its corresponding specificity and were reported as the median (2.5–97.5 percentile). We also extensively analysed the attention behaviours on different levels to infer what, how and when different clinical concepts contribute to the classification.

The source codes detailing the network architecture and hyperparameters are available at: [https://github.com/aaq109/CareNet](https://github.com/aaq109/CARENET)
